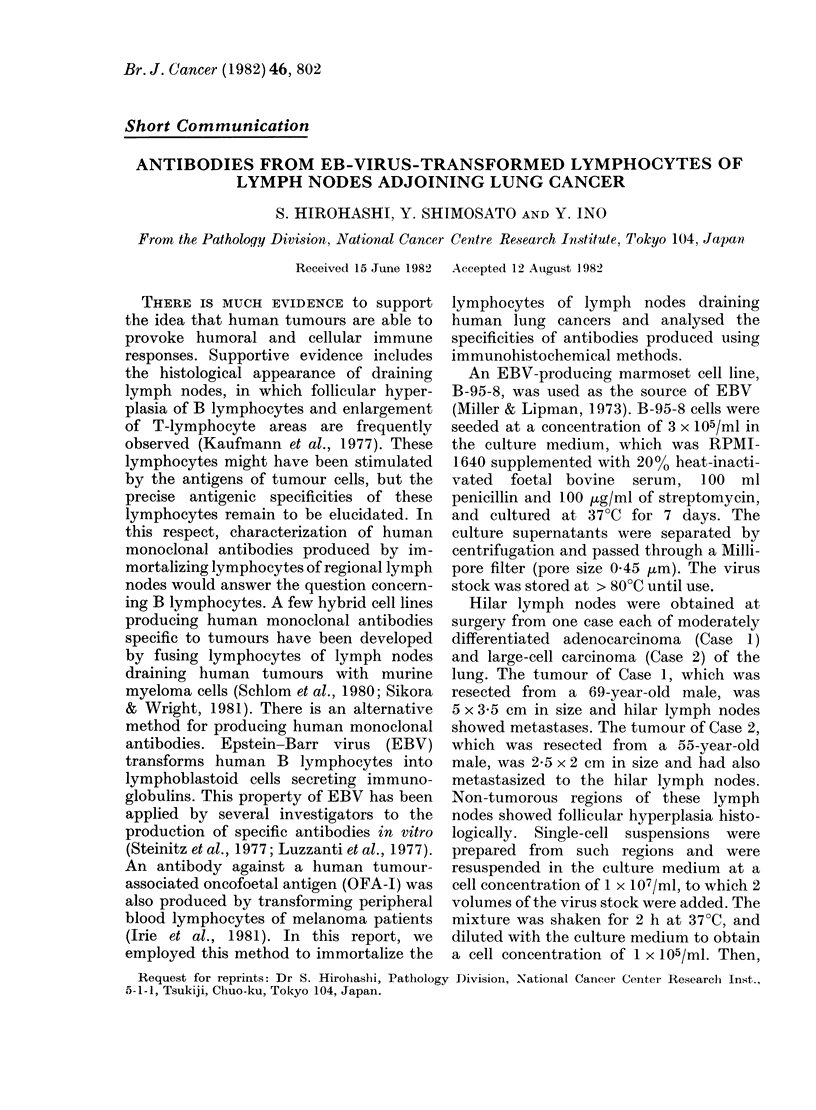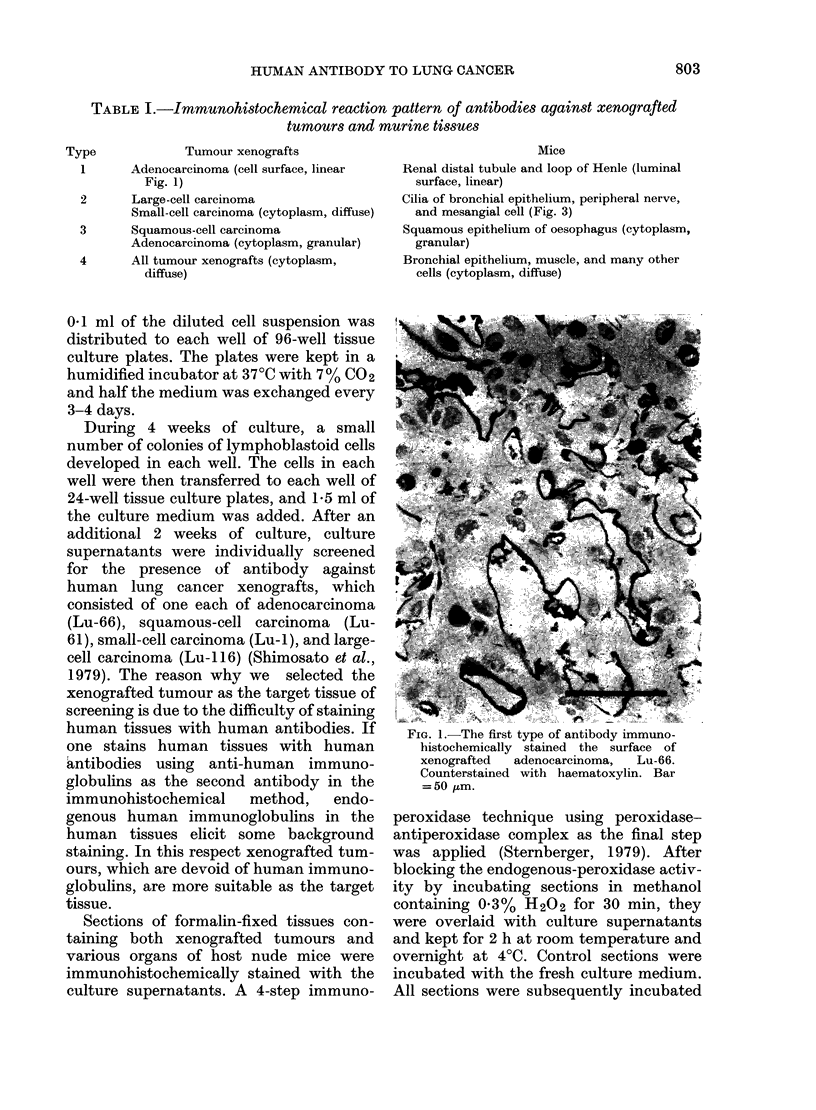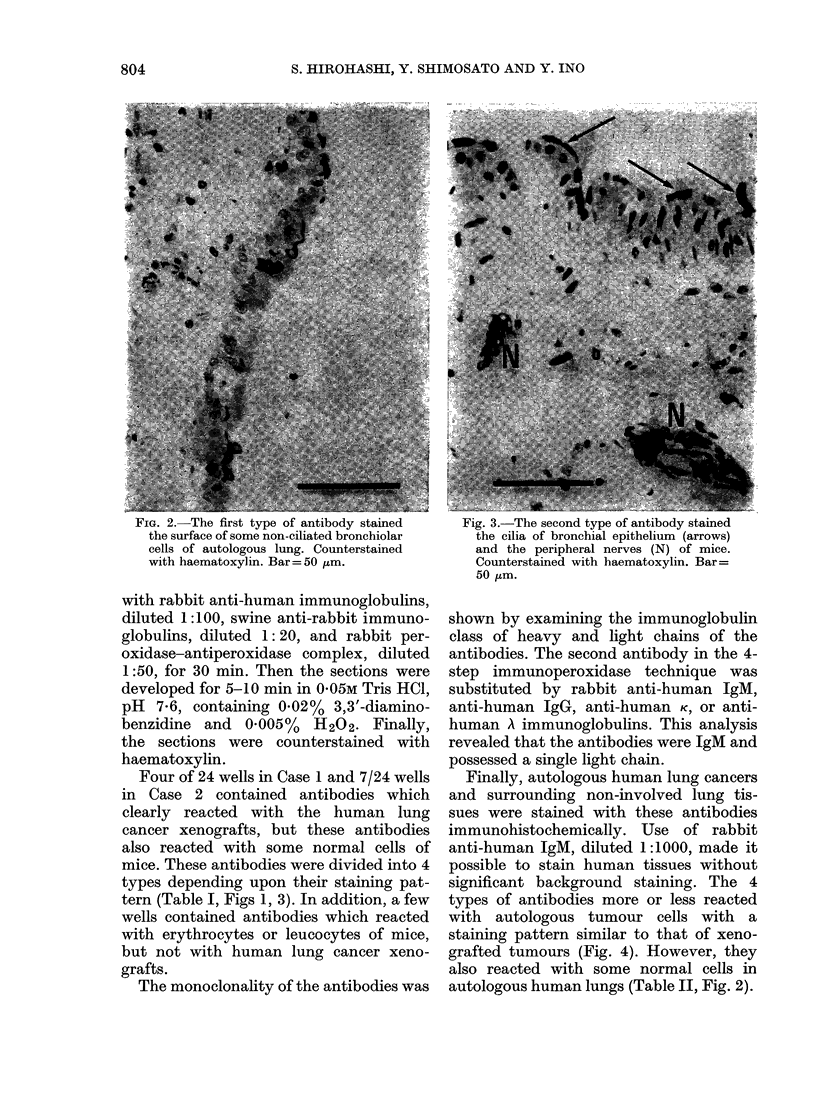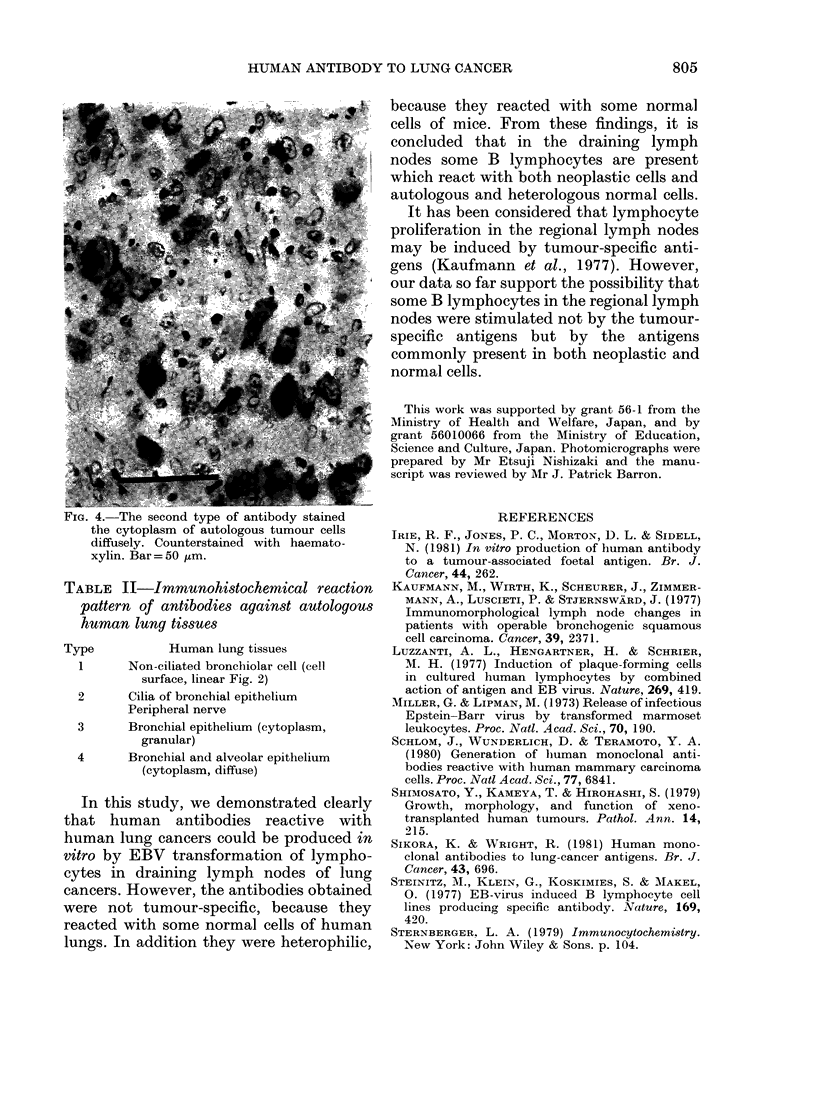# Antibodies from EB-virus-transformed lymphocytes of lymph nodes adjoining lung cancer.

**DOI:** 10.1038/bjc.1982.273

**Published:** 1982-11

**Authors:** S. Hirohashi, Y. Shimosato, Y. Ino

## Abstract

**Images:**


*Br. J. Cancer* (1982) **46**, 802

***Short Communication***

### ANTIBODIES FROM EB-VIRUS-TRANSFORMED LYMPHOCYTES OF LYMPH NODES ADJOINING LUNG CANCER

S. HIROHASHI, Y. SHIMOSATO AND Y. INO

*From the Pathology Division, National Cancer Centre Research Institute, Tokyo* 104, *Japan*

Received 15 June 1982 Accepted 12 August 1982

THERE IS MUCH EVIDENCE to support the idea that human tumours are able to provoke humoral and cellular immune responses. Supportive evidence includes the histological appearance of draining lymph nodes, in which follicular hyperplasia of B lymphocytes and enlargement of T-lymphocyte areas are frequently observed (Kaufmann *et al.*, 1977). These lymphocytes might have been stimulated by the antigens of tumour cells, but the precise antigenic specificities of these lymphocytes remain to be elucidated. In this respect, characterization of human monoclonal antibodies produced by immortalizing lymphocytes of regional lymph nodes would answer the question concerning B lymphocytes. A few hybrid cell lines producing human monoclonal antibodies specific to tumours have been developed by fusing lymphocytes of lymph nodes draining human tumours with murine myeloma cells (Schlom *et al.*, 1980; Sikora & Wright, 1981). There is an alternative method for producing human monoclonal antibodies. Epstein–Barr virus (EBV) transforms human B lymphocytes into lymphoblastoid cells secreting immunoglobulins. This property of EBV has been applied by several investigators to the production of specific antibodies *in vitro* (Steinitz *et al.*, 1977; Luzzanti *et al.*, 1977). An antibody against a human tumour-associated oncofoetal antigen (OFA-I) was also produced by transforming peripheral blood lymphocytes of melanoma patients (Irie *et al.*, 1981). In this report, we employed this method to immortalize the lymphocytes of lymph nodes draining human lung cancers and analysed the specificities of antibodies produced using immunohistochemical methods.

An EBV-producing marmoset cell line, B-95-8, was used as the source of EBV (Miller & Lipman, 1973). B-95-8 cells were seeded at a concentration of $3 \times 10^5$/ml in the culture medium, which was RPMI-1640 supplemented with 20% heat-inactivated foetal bovine serum, 100 ml penicillin and 100 $\mu$g/ml of streptomycin, and cultured at 37°C for 7 days. The culture supernatants were separated by centrifugation and passed through a Millipore filter (pore size 0·45 $\mu$m). The virus stock was stored at > 80°C until use.

Hilar lymph nodes were obtained at surgery from one case each of moderately differentiated adenocarcinoma (Case 1) and large-cell carcinoma (Case 2) of the lung. The tumour of Case 1, which was resected from a 69-year-old male, was 5 × 3·5 cm in size and hilar lymph nodes showed metastases. The tumour of Case 2, which was resected from a 55-year-old male, was 2·5 × 2 cm in size and had also metastasized to the hilar lymph nodes. Non-tumorous regions of these lymph nodes showed follicular hyperplasia histologically. Single-cell suspensions were prepared from such regions and were resuspended in the culture medium at a cell concentration of $1 \times 10^7$/ml, to which 2 volumes of the virus stock were added. The mixture was shaken for 2 h at 37°C, and diluted with the culture medium to obtain a cell concentration of $1 \times 10^5$/ml. Then,

Request for reprints: Dr S. Hirohashi, Pathology Division, National Cancer Center Research Inst., 5-1-1, Tsukiji, Chuo-ku, Tokyo 104, Japan.

TABLE I.—*Immunohistochemical reaction pattern of antibodies against xenografted tumours and murine tissues*

| Type | Tumour xenografts | Mice |
|---|---|---|
| 1 | Adenocarcinoma (cell surface, linear Fig. 1) | Renal distal tubule and loop of Henle (luminal surface, linear) |
| 2 | Large-cell carcinoma<br>Small-cell carcinoma (cytoplasm, diffuse) | Cilia of bronchial epithelium, peripheral nerve, and mesangial cell (Fig. 3) |
| 3 | Squamous-cell carcinoma<br>Adenocarcinoma (cytoplasm, granular) | Squamous epithelium of oesophagus (cytoplasm, granular) |
| 4 | All tumour xenografts (cytoplasm, diffuse) | Bronchial epithelium, muscle, and many other cells (cytoplasm, diffuse) |

0·1 ml of the diluted cell suspension was distributed to each well of 96-well tissue culture plates. The plates were kept in a humidified incubator at 37°C with 7% $CO_2$ and half the medium was exchanged every 3–4 days.

During 4 weeks of culture, a small number of colonies of lymphoblastoid cells developed in each well. The cells in each well were then transferred to each well of 24-well tissue culture plates, and 1·5 ml of the culture medium was added. After an additional 2 weeks of culture, culture supernatants were individually screened for the presence of antibody against human lung cancer xenografts, which consisted of one each of adenocarcinoma (Lu-66), squamous-cell carcinoma (Lu-61), small-cell carcinoma (Lu-1), and large-cell carcinoma (Lu-116) (Shimosato *et al.*, 1979). The reason why we selected the xenografted tumour as the target tissue of screening is due to the difficulty of staining human tissues with human antibodies. If one stains human tissues with human antibodies using anti-human immunoglobulins as the second antibody in the immunohistochemical method, endogenous human immunoglobulins in the human tissues elicit some background staining. In this respect xenografted tumours, which are devoid of human immunoglobulins, are more suitable as the target tissue.

Sections of formalin-fixed tissues containing both xenografted tumours and various organs of host nude mice were immunohistochemically stained with the culture supernatants. A 4-step immuno-

FIG. 1.—The first type of antibody immunohistochemically stained the surface of xenografted adenocarcinoma, Lu-66. Counterstained with haematoxylin. Bar = 50 μm.

peroxidase technique using peroxidase–antiperoxidase complex as the final step was applied (Sternberger, 1979). After blocking the endogenous-peroxidase activity by incubating sections in methanol containing 0·3% $H_2O_2$ for 30 min, they were overlaid with culture supernatants and kept for 2 h at room temperature and overnight at 4°C. Control sections were incubated with the fresh culture medium. All sections were subsequently incubated

FIG. 2.—The first type of antibody stained the surface of some non-ciliated bronchiolar cells of autologous lung. Counterstained with haematoxylin. Bar = 50 μm.

Fig. 3.—The second type of antibody stained the cilia of bronchial epithelium (arrows) and the peripheral nerves (N) of mice. Counterstained with haematoxylin. Bar = 50 μm.

with rabbit anti-human immunoglobulins, diluted 1:100, swine anti-rabbit immunoglobulins, diluted 1:20, and rabbit peroxidase–antiperoxidase complex, diluted 1:50, for 30 min. Then the sections were developed for 5–10 min in 0·05M Tris HCl, pH 7·6, containing 0·02% 3,3′-diaminobenzidine and 0·005% $H_2O_2$. Finally, the sections were counterstained with haematoxylin.

Four of 24 wells in Case 1 and 7/24 wells in Case 2 contained antibodies which clearly reacted with the human lung cancer xenografts, but these antibodies also reacted with some normal cells of mice. These antibodies were divided into 4 types depending upon their staining pattern (Table I, Figs 1, 3). In addition, a few wells contained antibodies which reacted with erythrocytes or leucocytes of mice, but not with human lung cancer xenografts.

The monoclonality of the antibodies was shown by examining the immunoglobulin class of heavy and light chains of the antibodies. The second antibody in the 4-step immunoperoxidase technique was substituted by rabbit anti-human IgM, anti-human IgG, anti-human κ, or anti-human λ immunoglobulins. This analysis revealed that the antibodies were IgM and possessed a single light chain.

Finally, autologous human lung cancers and surrounding non-involved lung tissues were stained with these antibodies immunohistochemically. Use of rabbit anti-human IgM, diluted 1:1000, made it possible to stain human tissues without significant background staining. The 4 types of antibodies more or less reacted with autologous tumour cells with a staining pattern similar to that of xenografted tumours (Fig. 4). However, they also reacted with some normal cells in autologous human lungs (Table II, Fig. 2).

FIG. 4.—The second type of antibody stained the cytoplasm of autologous tumour cells diffusely. Counterstained with haematoxylin. Bar = 50 μm.

TABLE II—*Immunohistochemical reaction pattern of antibodies against autologous human lung tissues*

| Type | Human lung tissues |
|---|---|
| 1 | Non-ciliated bronchiolar cell (cell surface, linear Fig. 2) |
| 2 | Cilia of bronchial epithelium<br>Peripheral nerve |
| 3 | Bronchial epithelium (cytoplasm, granular) |
| 4 | Bronchial and alveolar epithelium (cytoplasm, diffuse) |

In this study, we demonstrated clearly that human antibodies reactive with human lung cancers could be produced *in vitro* by EBV transformation of lymphocytes in draining lymph nodes of lung cancers. However, the antibodies obtained were not tumour-specific, because they reacted with some normal cells of human lungs. In addition they were heterophilic, because they reacted with some normal cells of mice. From these findings, it is concluded that in the draining lymph nodes some B lymphocytes are present which react with both neoplastic cells and autologous and heterologous normal cells.

It has been considered that lymphocyte proliferation in the regional lymph nodes may be induced by tumour-specific antigens (Kaufmann *et al.*, 1977). However, our data so far support the possibility that some B lymphocytes in the regional lymph nodes were stimulated not by the tumour-specific antigens but by the antigens commonly present in both neoplastic and normal cells.

This work was supported by grant 56-1 from the Ministry of Health and Welfare, Japan, and by grant 56010066 from the Ministry of Education, Science and Culture, Japan. Photomicrographs were prepared by Mr Etsuji Nishizaki and the manuscript was reviewed by Mr J. Patrick Barron.

### REFERENCES

IRIE, R. F., JONES, P. C., MORTON, D. L. & SIDELL, N. (1981) *In vitro* production of human antibody to a tumour-associated foetal antigen. *Br. J. Cancer*, **44**, 262.

KAUFMANN, M., WIRTH, K., SCHEURER, J., ZIMMERMANN, A., LUSCIETI, P. & STJERNSWÄRD, J. (1977) Immunomorphological lymph node changes in patients with operable bronchogenic squamous cell carcinoma. *Cancer*, **39**, 2371.

LUZZANTI, A. L., HENGARTNER, H. & SCHRIER, M. H. (1977) Induction of plaque-forming cells in cultured human lymphocytes by combined action of antigen and EB virus. *Nature*, **269**, 419.

MILLER, G. & LIPMAN, M. (1973) Release of infectious Epstein–Barr virus by transformed marmoset leukocytes. *Proc. Natl. Acad. Sci.*, **70**, 190.

SCHLOM, J., WUNDERLICH, D. & TERAMOTO, Y. A. (1980) Generation of human monoclonal antibodies reactive with human mammary carcinoma cells. *Proc. Natl Acad. Sci.*, **77**, 6841.

SHIMOSATO, Y., KAMEYA, T. & HIROHASHI, S. (1979) Growth, morphology, and function of xenotransplanted human tumours. *Pathol. Ann.* **14**, 215.

SIKORA, K. & WRIGHT, R. (1981) Human monoclonal antibodies to lung-cancer antigens. *Br. J. Cancer*, **43**, 696.

STEINITZ, M., KLEIN, G., KOSKIMIES, S. & MAKEL, O. (1977) EB-virus induced B lymphocyte cell lines producing specific antibody. *Nature*, **169**, 420.

STERNBERGER, L. A. (1979) *Immunocytochemistry.* New York: John Wiley & Sons. p. 104.